# Engineering alginate hydrogels loaded with resveratrol as a dual platform for cancer therapy and wound regeneration

**DOI:** 10.1186/s11671-026-04928-5

**Published:** 2026-10-02

**Authors:** Yang Fu, Yuanxin Ge, Shixiong Yi, Qifeng Peng, Heng Jiang, Jie Zhou, Chengxuan Liu

**Affiliations:** 1 Department of Rehabilitation, Chongqing Traditional Chinese Medicine Hospital, Chongqing, 400021 China; 2 The First Affiliated Hospital of Chongqing College of Chinese Medicine, Chongqing, 400021 China

**Keywords:** Resveratrol, Alginate, Hydrogel, Cancer therapy, Wound healing, Drug delivery

## Abstract

Hydrogels represent a highly adaptable biomaterial platform for a broad range of biomedical uses, largely because they can replicate the extracellular matrix while retaining substantial amounts of aqueous fluid. Among the polymers employed for hydrogel fabrication, naturally derived macromolecules are particularly appealing thanks to their favorable biocompatibility and biodegradation profiles. Alginate, in particular, has drawn considerable attention as a matrix-forming polymer owing to its wide natural availability, low cost, and the relative ease with which it can be crosslinked into three-dimensional networks. Meanwhile, the therapeutic management of malignancies and non-healing wounds continues to impose considerable burdens on clinical and pharmaceutical practice. Resveratrol (RSV), a polyphenolic stilbene, has shown encouraging outcomes in addressing both conditions; nevertheless, its translation into the clinic is hampered by intrinsic drawbacks including limited solubility in aqueous media, marked hydrophobic character, and rapid elimination following administration. The present article surveys recent progress on RSV-loaded alginate hydrogel systems developed for oncological and wound-repair indications. Particular attention is devoted to the molecular mechanisms underlying RSV action and to its intracellular disposition. Subsequently, the review details the principal fabrication strategies used to incorporate RSV within alginate networks, together with the structural and environmental variables that modulate drug release. The discussion closes by highlighting avenues for improving therapeutic performance and outlining forward-looking strategies for engineering next-generation RSV-alginate hydrogel platforms with broadened application scope. Distinct from previous reviews, this work is organized around the structure–function relationships that govern RSV bioavailability within alginate matrices, and it treats the two therapeutic fronts—oncology and wound repair—where alginate-based delivery offers the greatest translational leverage in an integrated framework. The manuscript is intended for formulation scientists, biomaterials engineers, and translational researchers seeking practical design guidelines rather than a catalogue of individual studies. Specifically, readers will find (i) comparative analysis of encapsulation strategies with their quantitative performance metrics, (ii) explicit linkage between hydrogel network parameters (crosslinking density, mesh size, porosity) and in vivo therapeutic outcomes, and (iii) a critical assessment of the barriers—scalability, regulatory harmonization, long-term safety, and batch reproducibility—that currently separate bench-scale performance from clinical translation.

## Introduction

The design of innovative delivery vehicles for pharmaceutically significant biomolecules represents a pivotal research focus in contemporary biomedical science. Polysaccharides have emerged as prominent candidates due to their capacity to confer controlled material properties and synergistic interactions with diverse therapeutic agents. Among these biopolymers, alginate has been extensively investigated for fabricating hydrogel matrices, owing to its exceptional biocompatibility, abundance, and facile gelation under physiological conditions [1].

Cancer and chronic wound management remain formidable challenges for pharmaceutical and healthcare sectors. Resveratrol (RSV) has demonstrated significant therapeutic efficacy in both contexts. It exhibits pleiotropic anticancer mechanisms targeting carcinogenesis stages, including initiation, promotion, and progression. Concurrently, RSV confers beneficial effects in wound healing through potent antioxidant and anti-inflammatory properties, effectively scavenging reactive oxygen species (ROS) and mitigating oxidative stress [2].

However, the clinical translation of RSV is severely constrained by inherent limitations, such as suboptimal aqueous solubility, pronounced photosensitivity, and rapid metabolic degradation. These factors result in minimal bioavailability, often attributable to extensive first-pass metabolism and accelerated systemic clearance. Consequently, over 70% of orally administered RSV undergoes rapid excretion, necessitating advanced delivery systems to enhance stability and absorption [3, 4]. The development of alginate-based hydrogels has arisen as a viable strategy to address these deficiencies. Encapsulation within hydrogel matrices enhances stability, improves bioavailability, and enables controlled release at targeted therapeutic sites. RSV-loaded alginate hydrogels have garnered considerable interest for applications in oncology and regenerative medicine, facilitated by ease of functional modification to tailor drug delivery profiles [5, 6]. This review comprehensively explores the research cascade surrounding RSV-loaded alginate hydrogel matrices for cancer therapy and wound healing. Emphasis is placed on elucidating the mechanistic effects of RSV and its intracellular fate. Additionally, the review discusses three principal design categories for these hydrogels—covalent conjugation, electrostatic interactions, and physical entrapment—alongside the structural and environmental parameters governing release behavior. Finally, key aspects for enhancing hydrogel efficacy and novel strategies for developing multifunctional RSV-alginate systems are highlighted.

Despite the growing body of literature on RSV delivery systems, several critical gaps remain unaddressed in existing reviews. First, most prior reviews treat cancer therapy and wound healing in isolation, despite the fact that both pathologies share overlapping molecular drivers—chronic oxidative stress, dysregulated NF-κB signaling, and aberrant angiogenesis—that can be jointly targeted by a single well-engineered platform. Second, while individual encapsulation strategies have been described extensively, quantitative side-by-side comparisons of encapsulation efficiency, release kinetics, particle dimensions, and in vivo therapeutic outcomes across hydrogel formats are rarely consolidated, limiting the ability of formulation scientists to benchmark new designs. Third, the mechanistic link between hydrogel design parameters and downstream biological responses is typically described qualitatively, providing limited rational design guidance. Fourth, the translational hurdles that separate promising preclinical outcomes from clinical adoption—manufacturing scalability, regulatory alignment, long-term toxicity of crosslinkers and residual solvents, and batch-to-batch reproducibility—have received comparatively little dedicated attention in the existing review literature.

The present review is structured to address these gaps directly. Following a concise overview of RSV’s molecular pharmacology and cellular fate, we systematically compare these three aforementioned encapsulation strategies and analyze the intrinsic and extrinsic variables that govern RSV release from alginate networks, culminating in an explicit synthesis of how hydrogel design parameters map onto in vivo biological outcomes. We then critically survey representative applications in wound repair and oncology with consolidated quantitative performance benchmarks. A dedicated discussion of clinical translation challenges precedes the final synthesis of design principles and forward-looking strategies. The manuscript is therefore intended not as an exhaustive catalogue of formulations but as a practical, critically curated reference for biomaterials engineers and formulation scientists seeking evidence-based design criteria for next-generation RSV–alginate platforms.

## Biological properties of resveratrol

### Mechanism of resveratrol

#### Cancer biology

RSV exhibits pleiotropic anticancer mechanisms targeting multiple carcinogenic stages, including initiation, promotion, and progression [7]. This compound modulates key signaling pathways, such as PI3K/AKT, MAPK, and Wnt/β-catenin, to inhibit cancer cell proliferation, induce apoptosis, arrest the cell cycle, suppress angiogenesis, and impede metastasis [8, 9]. Critical mechanisms encompass the downregulation of pro-inflammatory transcription factors like NF-κB, inhibition of cyclooxygenase (COX) enzymes, and epigenetic regulation involving histone acetylation and DNA methylation [10]. Furthermore, RSV activates tumor suppressor genes, particularly p53, contributing to its chemopreventive and therapeutic efficacy against various cancers [11].

#### Wound healing

Wound healing constitutes a complex, multi-phase process comprising hemostasis, inflammation, proliferation, and tissue remodeling [12]. RSV confers beneficial effects across these phases, primarily through its potent antioxidant and anti-inflammatory properties, which scavenge ROS and mitigate oxidative stress [13]. During the inflammatory phase, it downregulates pro-inflammatory cytokines like TNF-α and IL-6, thereby optimizing immune responses [14]. In the proliferative phase, RSV promotes angiogenesis via upregulation of vascular endothelial growth factor (VEGF) and enhances fibroblast proliferation and migration [15]. At the fibroblast level, this regenerative activity also involves suppression of excessive Notch signaling under hyperglycemic conditions. Inhibition of this pathway restores dermal fibroblast proliferation and migration that are otherwise impaired by high glucose, thereby linking a defined intracellular target to improved granulation-tissue formation and wound closure in diabetes [16]. It also facilitates the remodeling phase by stimulating collagen synthesis and deposition, which improves the tensile strength of healed tissue [17]. Additionally, its inherent antimicrobial activity reduces infection risks, rendering it a viable candidate for managing both acute and chronic wounds [18] .

### Molecular targets of resveratrol

RSV is a multifunctional phytochemical that directly interacts with diverse molecular targets to modulate critical cellular processes. Over 20 distinct biomolecules have been identified as direct binding partners, including transcription factors like NF-κB, p53, inflammation-associated enzymes like COX-2, iNOS, signaling kinases like AKT, ERK, AMPK, and apoptosis regulators like Bcl-2 family proteins, cyclins [19]. Notably, RSV activates sirtuins (particularly SIRT1), thereby influencing epigenetic modifications and cellular senescence. It also functions as a selective estrogen receptor modulator (SERM), demonstrating therapeutic potential in hormone-dependent pathologies [20]. Recent studies reveal interactions with non-canonical targets such as DEAD-box RNA helicase DDX5, suppressing mTORC1 signaling in cancer cells [21], and novel proteins identified through inverse molecular docking like SRC, PIK3CA [22]. Additionally, its metal-chelating capacity for divalent ions (Cu²⁺, Fe²⁺) contributes to antioxidant effects and modulates metalloenzyme activity [23].

#### Targeting enzymatic and signaling pathways to restrict cancer growth and promote healing

RSV modulates a broad spectrum of enzymatic and signaling pathways critical for inhibiting carcinogenesis and facilitating tissue regeneration. Key targets include cyclooxygenase-2 (COX-2) and inducible nitric oxide synthase (iNOS), enzymes overexpressed in tumors and chronic inflammatory conditions; RSV suppresses their expression and activity, thereby reducing pro-inflammatory prostaglandins and nitric oxide production [24]. This suppression contributes significantly to its chemopreventive and anti-inflammatory properties. Additionally, RSV activates sirtuins, particularly SIRT1, an NAD^+^-dependent deacetylase associated with longevity and metabolic regulation. SIRT1 activation influences downstream processes such as apoptosis, cellular stress response, and mitochondrial biogenesis, thereby mitigating cancer progression [25]. Furthermore, RSV enhances the activity of AMP-activated protein kinase (AMPK), a central regulator of cellular energy homeostasis. AMPK activation inhibits anabolic pathways including fatty acid and protein synthesis, restricting metabolic plasticity in cancer cells [26].

RSV also exerts potent antioxidant effects by upregulating endogenous enzymes like superoxide dismutase (SOD) and catalase, while inhibiting pro-oxidant enzymes such as NADPH oxidase [27]. In cell survival regulation, RSV inhibits the Akt/mTOR signaling pathway, a driver of cell growth frequently dysregulated in cancer, leading to reduced protein synthesis and induction of autophagy and apoptosis [28]. Moreover, it interferes with the Wnt/β-catenin pathway by promoting β-catenin degradation and downregulating oncogenic targets like c-Myc and cyclin D1, thereby suppressing proliferation and inducing cell cycle arrest [29]. Through this multi-targeted approach, RSV creates a molecular environment conducive to restricting tumor growth and promoting wound resolution, demonstrating synergistic effects in both cancer therapy and regenerative applications, as evidenced in preclinical models of hydrogel-based delivery [30].

#### Core pathways of anti-inflammatory and antioxidant effects

RSV exerts significant anti-inflammatory and antioxidant effects primarily through the modulation of key signaling pathways, which constitute the fundamental mechanisms for its therapeutic efficacy in both cancer therapy and wound healing contexts.

##### Inhibition of the NF-κB pathway

The NF-κB signaling pathway functions as a central regulator of inflammatory responses and is intimately linked to tumorigenesis and chronic wound progression. RSV effectively suppresses NF-κB activation by inhibiting the phosphorylation and degradation of its inhibitory protein, IκBα, thereby blocking the nuclear translocation of the p50/p65 heterodimer. This inhibition leads to a downregulation of multiple pro-inflammatory mediators, including TNF-α, IL-1β, IL-6, chemokines, adhesion molecules, and enzymes such as COX-2 and iNOS, which are critical in perpetuating inflammatory cascades [31]. By attenuating NF-κB-driven inflammatory responses, RSV alleviates tissue damage and promotes resolution in chronic wounds, while simultaneously curbing the inflammatory microenvironment that facilitates tumor growth and metastasis [32].

##### Activation of the Nrf2 pathway

The Nrf2-ARE pathway serves as a primary cellular defense mechanism against oxidative and electrophilic stress. RSV activates Nrf2 signaling by facilitating its dissociation from the cytosolic repressor Keap1, thereby promoting Nrf2 nuclear translocation. Upon entering the nucleus, Nrf2 binds to the Antioxidant Response Element (ARE), initiating transcription of cytoprotective genes. These include genes encoding phase II detoxifying enzymes, such as NAD(P)H: quinone oxidoreductase 1 (NQO1) and glutathione S-transferase (GST), alongside key antioxidant proteins including heme oxygenase-1 (HO-1), superoxide dismutase (SOD), and catalase. Through this mechanism, RSV enhances cellular antioxidant capacity, reduces excessive ROS accumulation, and mitigates oxidative damage—effects critical for suppressing carcinogenesis and establishing a redox-balanced microenvironment conducive to wound healing [33, 34]. Recent studies further demonstrate that RSV-mediated Nrf2 activation promotes mitochondrial function and accelerates diabetic wound repair by upregulating detoxification enzymes and reducing lipid peroxidation [35]. Beyond this antioxidant axis, RSV also accelerates diabetic wound angiogenesis by suppressing endothelial ferroptosis through the PI3K–AKT–Nrf2 pathway, linking its redox activity to functional vascular regeneration [32].

##### Inhibition of the JAK-STAT pathway

The JAK-STAT pathway, particularly its STAT3 branch, is a central mediator of inflammatory signaling and is aberrantly activated in chronic inflammatory diseases and cancers. RSV suppresses JAK-STAT signaling by inhibiting phosphorylation of JAK1 and JAK2, thereby preventing STAT protein activation and dimerization, with notable efficacy against STAT3 [36]. This inhibition attenuates the production of pro-inflammatory cytokines like TNF-α, IL-6 and growth factors, resolving excessive immune activation and mitigating tissue damage in chronic wounds. Additionally, RSV disrupts the cytokine network that supports tumor survival and immune evasion, partly through downregulating STAT3 nuclear translocation and its downstream targets [37]. In cancer therapy, this mechanism reduces metastasis and chemoresistance in models such as osteosarcoma and non-small-cell lung cancer [38]. The JAK2/STAT3 node also connects RSV activity to inflammasome-dependent inflammation within the tumor microenvironment. Suppression of JAK2 and STAT3 phosphorylation limits STAT3 nuclear translocation and reduces NLRP3 inflammasome activation, accompanied by lower expression of IL-1β, IL-18, IL-6, and TNF-α in glioblastoma cells. This mechanism broadens the functional consequences of JAK2/STAT3 inhibition from direct control of tumor-cell survival to attenuation of the inflammatory conditions that support invasion and malignant progression [39]. Incomplete suppression of JAK2/STAT3 signaling may also permit adaptation to single-agent treatment. Combining RSV with a pharmacological JAK2 inhibitor intensifies pathway blockade and restores growth inhibition and apoptosis in glioblastoma cells that respond incompletely to either intervention alone. These findings support the use of localized co-delivery systems to maintain both agents within the tumor while limiting systemic exposure, although the optimal concentration ratio and release sequence remain to be established [40].

#### Core mechanisms of inhibiting proliferation and promoting apoptosis

RSV exerts potent antitumor effects by precisely modulating cell cycle progression, apoptosis-related proteins, and oncogenic signaling pathways, thereby suppressing tumor growth and inducing programmed cell death [41, 42].

##### Regulation of cell cycle checkpoint proteins

RSV induces cell cycle arrest predominantly at the G1/S or G2/M phases via dysregulation of cyclins, cyclin-dependent kinases (CDKs), and CDK inhibitors. It downregulates cyclins such as cyclin D1 and E, and CDKs including CDK4 and CDK2, while upregulating CDK inhibitors p21^Cip1/Waf1^ and p27^Kip1^. This disruption impedes active cyclin-CDK complex formation, halting cell cycle progression and curtailing uncontrolled cancer cell proliferation [43, 44].

##### Pro-apoptotic effects: modulation of bcl-2 family proteins

RSV promotes apoptosis by shifting the equilibrium between pro- and anti-apoptotic Bcl-2 family members. It suppresses anti-apoptotic proteins like Bcl-2 and Bcl-xL and upregulates/activates pro-apoptotic proteins including Bax and Bak. This imbalance triggers mitochondrial outer membrane permeabilization (MOMP), cytochrome c release, caspase cascade activation, and ultimate apoptotic cell death—a central mechanism in RSV’s efficacy against malignant cells [45, 46].

##### Tumor suppressor pathway: activation of p53 signaling

RSV activates the p53 tumor suppressor pathway primarily through post-translational modifications that stabilize the p53 protein and enhance its transcriptional activity. This stabilization induces the transactivation of p53 target genes, including the cyclin-dependent kinase inhibitor p21, which mediates G1/S phase arrest, and pro-apoptotic factors such as Bax, Puma, and Noxa, which promote mitochondrial apoptosis. Consequently, RSV effectively eliminates genomically damaged or malignant cells, thereby exerting potent anticancer effects [47]. Notably, recent studies indicate that RSV directly interacts with p53’s DNA-binding domain to prevent amyloid-like aggregation of mutant p53 proteins, restoring wild-type tumor-suppressive functions [48]. Additionally, it epigenetically reactivates p53 expression by modulating histone methylation including suppressing PRMT5-mediated H4R3me2s and acetylation marks including enhancing H3K9ac at the p53 promoter locus [49].

##### Inhibition of pro-proliferative pathways: Wnt/β-catenin and MAPK/ERK signaling

RSV coordinately suppresses two critical oncogenic cascades

###### Wnt/β-catenin signaling

By promoting phosphorylation-dependent degradation of β-catenin, RSV downregulates downstream oncogenes including c-Myc and cyclin D1 and inhibits epithelial-mesenchymal transition. This occurs via suppression of key effectors such as PRDX4 and SIX4/SPHK2, ultimately impeding tumor progression and metastasis[50–52].

###### MAPK/ERK signaling

RSV attenuates ERK1/2 phosphorylation by inhibiting upstream kinases including RAF and MEK. This suppresses proliferation and survival signals while synergizing with p53-mediated apoptosis. Hyperglycemia-driven ROS production, which activates ERK/p38 MAPK in pancreatic cancer, is also mitigated by RSV [53, 54].

The dual inhibition of these pathways disrupts cancer cell growth, invasiveness, and metabolic reprogramming, highlighting RSV’s multifaceted therapeutic potential.

#### Histone modifications: deacetylases and acetyltransferases

RSV significantly influences epigenetic regulation primarily via the modulation of histone-modifying enzymes, thereby altering gene expression profiles essential for cancer prevention and cellular homeostasis.

##### Histone deacetylases (HDACs)

Histone deacetylases catalyze the removal of acetyl groups from histone proteins, leading to chromatin condensation and transcriptional repression. RSV serves as a potent modulator of HDAC activity, specifically inhibiting Class I HDACs (e.g., HDAC1-HDAC3) in multiple cancer models, which results in increased histone acetylation and re-expression of silenced tumor suppressor genes [55, 56]. Conversely, it functions as a selective activator of sirtuins (Class III HDACs), notably SIRT1. While its role as a direct allosteric activator has been scrutinized due to potential assay artifacts, there is a growing consensus that RSV enhances SIRT1 activity via substrate-specific stabilization or indirect pathways, such as AMPK activation. This activation enhances deacetylation of target substrates such as p53, FOXO transcription factors, and PGC-1α, culminating in improved cell survival under stress, augmented mitochondrial biogenesis, and decelerated cellular senescence [49]. This dual regulation underscores RSV’s unique capacity to balance cellular fate decisions by simultaneously suppressing oncogenic pathways and promoting adaptive responses.

##### Histone acetyltransferases (HATs)

Histone acetyltransferases facilitate the addition of acetyl groups to lysine residues on histone tails, inducing chromatin relaxation and transcriptional activation. RSV demonstrates context-dependent effects on HAT activity: it inhibits specific HATs such as p300/CBP under hyperproliferative conditions to suppress oncogene expression, while paradoxically supporting HAT function in physiological contexts to maintain normal transcriptional programs. Such selective modulation aids in normalizing aberrant epigenetic landscapes without disrupting critical cellular functions, contributing to RSV’s chemopreventive properties by restoring balanced gene expression in pre-malignant cells [57]. However, comprehensive signaling cascades governing this duality remain incompletely characterized due to limited mechanistic data in current literature; although evidence suggests involvement in NF-κB pathways, key intermediates like AKT require further validation [58].

#### DNA methyltransferases

DNA methylation serves as a fundamental epigenetic mechanism for gene silencing, with its dysregulation recognized as a hallmark of cancer pathogenesis. RSV directly targets DNA methyltransferases (DNMTs), the enzymes responsible for establishing and maintaining DNA methylation patterns. Empirical studies demonstrate that RSV inhibits DNMT1—the primary maintenance methyltransferase—through dual mechanisms of direct enzymatic interaction and transcriptional downregulation [59]. This inhibition induces global DNA hypomethylation and promotes site-specific demethylation of promoter regions in tumor suppressor genes (e.g., BRCA1, RARβ2, and GSTP1), thereby reactivating their transcriptional expression [60]. Consequently, the restoration of these silenced genes enhances RSV’s capacity to reinstate normal cellular growth control and apoptosis sensitivity in malignant cells.

Furthermore, RSV’s modulation of DNA methylation operates synergistically with its influence on histone modifications like acetylation and methylation. This coordinated epigenetic reprogramming amplifies its therapeutic efficacy across diverse pathologies, including cancer chemoprevention and age-related disorders characterized by epigenetic dysregulation [61]. Recent evidence further indicates that combinatorial approaches—such as RSV conjugated with pterostilbene or proanthocyanidins—synergistically inhibit DNMT activity and enhance tumor suppression through epigenetic pathway crosstalk [62].

### Pharmacokinetics and metabolic fate of resveratrol

#### Oral administration

Following oral ingestion, RSV is primarily absorbed in the duodenum via passive diffusion. However, it undergoes extensive first-pass metabolism in the intestinal epithelium and liver, resulting in rapid biotransformation [63]. Within enterocytes, phase II enzymes—notably sulfotransferases (SULTs) and uridine 5’-diphospho-glucuronosyltransferases (UGTs, such as UGT1A1 and UGT1A9)—efficiently conjugate RSV to form glucuronide and sulfate metabolites [64]. These conjugated derivatives dominate systemic circulation, while free RSV concentrations remain negligible (nanomolar range), even at high doses [65].

The gut microbiota further contributes to RSV metabolism, reducing it to dihydroresveratrol and generating novel metabolites like lunularin and 4-hydroxydibenzyl through dehydroxylation [66, 67]. Such metabolic pathways exhibit significant interindividual variability, with distinct “metabotypes” observed in human populations [68]. Crucially, efflux transporters—including P-glycoprotein (P-gp) and multidrug resistance-associated proteins (MRPs)—expressed on enterocyte apical membranes actively pump RSV and its metabolites back into the intestinal lumen, limiting net absorption [69].

Consequently, > 70% of orally administered RSV is excreted in feces and urine as sulfate and glucuronide conjugates, with a short elimination half-life, typically ranging from 2 to 14 h [70]. This rapid clearance and minimal bioavailability severely constrain therapeutic efficacy, necessitating advanced delivery systems to enhance stability and absorption [71].

Taken together, clinical pharmacokinetic evidence confirms a consistent dissociation between gastrointestinal absorption and systemic exposure to unchanged RSV. Although RSV can be absorbed efficiently, circulating parent-drug concentrations remain limited by rapid glucuronidation and sulfation, with substantial heterogeneity arising from dose, formulation, food intake, participant characteristics, and sampling strategy. Parent-RSV concentrations alone may therefore underestimate the total biologically available pool, because circulating conjugates and their potential tissue deconjugation also contribute to intracellular exposure [72].

#### Systemic administration

Systemic administration routes circumvent the extensive first-pass metabolism inherent to oral delivery, thereby achieving significantly elevated initial plasma concentrations of unmetabolized RSV [73]. Nevertheless, rapid tissue distribution and systemic clearance persist, predominantly mediated by hepatic processes [74].

Upon entering systemic circulation, RSV distributes widely to target tissues. However, its inherent hydrophobicity limits solubility in aqueous plasma without appropriate formulation adjuvants, restricting effective biodistribution [75]. The liver serves as the primary site for systemic metabolism, where high expression of phase II enzymes—notably uridine diphosphate-glucuronosyltransferases (UGTs) and sulfotransferases (SULTs)—drives efficient conjugation into RSV glucuronides and sulfates [76]. Studies demonstrate that a substantial proportion of intravenously administered RSV appears in bile as conjugated metabolites within minutes, confirming active hepatobiliary excretion [77].

These hepatic conjugates, or those exported basolaterally from extrahepatic tissues, undergo renal elimination due to enhanced hydrophilicity. The kidneys efficiently filter these metabolites, leading to urinary excretion [78]. Although systemic administration enhances parent compound bioavailability relative to oral intake, rapid hepatic conjugation coupled with renal and biliary clearance results in a short circulatory half-life (typically < 1 h) and a constrained therapeutic window. This pharmacokinetic profile underscores the challenge of maintaining therapeutically effective concentrations of free RSV in target tissues [79].

Consequently, advanced delivery platforms such as alginate-based hydrogels are critically needed. These systems protect RSV from premature metabolism, prolong systemic exposure, and enable tissue-specific delivery. Recent studies validate that hydrogel encapsulation significantly reduces clearance rates—e.g., alginate/graphene nanoplatelet composites enhance RSV retention by 4.25-fold compared to free drug [80], while pH-responsive alginate-pectin hydrogels facilitate intestine-targeted release with > 90% encapsulation efficiency [81]. Such innovations address the limitations of conventional administration and potentiate efficacy in oncology and regenerative medicine applications [82].

## Encapsulation methodologies for resveratrol within hydrogel networks

RSV, a naturally derived polyphenolic compound, encounters substantial obstacles in clinical application owing to its inherent limitations, including suboptimal aqueous solubility, pronounced photosensitivity, and rapid metabolic degradation. Encapsulation within hydrogel matrices emerges as a viable approach to circumvent these deficiencies, thereby augmenting stability, enhancing bioavailability, and enabling controlled release at specific therapeutic sites [81]. Among various hydrogel systems, alginate-based formulations stand out as a premier platform, attributed to their exceptional biocompatibility, gentle gelation conditions under physiological settings, and ease of functional modification to tailor drug delivery profiles [83]. Multiple strategies have been devised to efficiently incorporate RSV into the three-dimensional architecture of alginate hydrogels, with principal categories encompassing covalent conjugation, electrostatic interactions, and physical entrapment. The judicious selection of an encapsulation methodology is critical, as it profoundly impacts drug loading capacity, release kinetics, and the overall therapeutic efficacy of the resultant formulations, particularly in oncology and regenerative medicine contexts [84].

### Covalent conjugation with hydrogels

Covalent conjugation constitutes a robust technique for immobilizing RSV within the hydrogel matrix, effectively mitigating premature burst release and protecting the labile compound from enzymatic or chemical degradation. This method entails the establishment of stable covalent bonds, primarily between the phenolic hydroxyl groups of RSV and reactive moieties on the polymer backbone, yielding chemical gels with superior structural integrity and prolonged stability [80].

A widely adopted procedure involves activating the carboxylate functionalities of alginate via carbodiimide-based chemistry, such as the EDC/NHS (1-ethyl-3-(3-dimethylaminopropyl)carbodiimide/N-hydroxysuccinimide) system, followed by nucleophilic attack from RSV’s hydroxyl groups to form ester linkages. This conjugation can precede gelation, generating RSV-grafted alginate polymers that are subsequently crosslinked with calcium ions to fabricate the hydrogel network. Release kinetics in such systems are predominantly regulated by the hydrolysis of these covalent bonds, which can be engineered to exhibit pH-sensitivity or enzyme-responsiveness. For instance, ester bonds demonstrate accelerated hydrolysis in the mildly acidic microenvironments characteristic of tumors or chronic wounds, facilitating site-specific drug liberation and targeted therapeutic action [85].

Furthermore, to refine control over release dynamics, RSV has been conjugated to hydrogels via stimuli-labile linkers. The incorporation of disulfide bonds, for example, enables specific drug release in response to elevated intracellular glutathione (GSH) concentrations, a hallmark of malignant cells, thus exploiting redox-sensitive mechanisms. This covalent integration strategy not only elevates encapsulation efficiency and loading capacity by securing the drug firmly within the network but also imparts sustained and localized release profiles [86]. Such attributes are indispensable for long-term cancer management and progressive wound healing applications, as they ensure therapeutic agents remain tethered to the delivery vehicle until reaching the intended action site, thereby amplifying therapeutic outcomes while minimizing off-target adverse effects.

### Electrostatic interaction

Electrostatic interaction represents a versatile and relatively mild approach for fabricating RSV-loaded alginate hydrogels, particularly advantageous for the development of in-situ forming delivery systems, such as those employed in localized cancer therapy or wound healing applications. This method exploits reversible ionic cross-linking between oppositely charged polyelectrolytes under physiological conditions, leveraging alginate’s inherent anionic properties as a natural polysaccharide rich in carboxyl groups. Upon combination with polycations—including chitosan, poly-L-lysine (PLL), or specific cationic surfactants—alginate spontaneously assembles into a hydrogel network through electrostatic forces [87].

The encapsulation of RSV via electrostatic interaction can be realized through multiple strategies. In a direct approach, RSV is dispersed or dissolved within an alginate solution, which is subsequently extruded into a bath containing cross-linking polycations. During gelation, the nascent hydrogel network physically entraps the drug molecules. Nevertheless, owing to RSV’s hydrophobicity, this simplistic method often yields suboptimal encapsulation efficiency and limited drug loading capacity. To mitigate this limitation, a more refined strategy involves pre-complexing RSV with charged nanocarriers, such as chitosan nanoparticles or cationic liposomes. These positively charged entities are then integrated with the alginate solution; robust electrostatic attractions between the carriers and anionic alginate chains facilitate their efficient incorporation into the hydrogel matrix during ionic cross-linking. This not only substantially boosts encapsulation efficiency and loading capacity for hydrophobic RSV but also establishes a multi-barrier system that finely modulates drug release [81]. The resultant release kinetics are governed by a synergistic interplay between diffusion from the nano-carriers and the degradation or swelling behavior of the hydrogel itself.

The simplicity of this technique, avoidance of harsh chemical reactions, and capacity for in-situ gel formation under physiological conditions make electrostatic interaction highly attractive for topical delivery systems. It supports targeted therapeutic applications in scenarios such as wound repair or localized tumor treatment, ensuring sustained drug release while maintaining biocompatibility and efficacy [88]. Furthermore, recent innovations in electrostatic methods demonstrate enhanced tunability for optimizing hydrogel properties, reinforcing the suitability of this approach in advanced biomedical contexts [89].

### Simple diffusion/partitioning

Simple diffusion and partitioning, also referred to as passive absorption or equilibrium loading, constitutes a fundamental physical strategy for encapsulating hydrophobic compounds such as RSV within pre-formed or nascent alginate hydrogel matrices. This method leverages differential solubility and partitioning behavior of the drug molecule between an external solvent phase and the aqueous/solid microenvironment of the hydrogel network, independent of specific chemical bonding or electrostatic interactions [81].

In standard protocols, pre-fabricated alginate hydrogel constructs, such as ionically cross-linked beads, discs, or membranes, are immersed in an RSV-saturated organic solvent like ethanol or dimethyl sulfoxide. During immersion, hydrogel swelling facilitates solvent infiltration and subsequent diffusion of dissolved drug molecules into the hydrophilic pores of its three-dimensional architecture. The encapsulation efficacy is governed by favorable partitioning of hydrophobic RSV into intrinsic hydrophobic microdomains within the polymer matrix, which may be inherent to low-polarity regions of alginate chains or deliberately engineered through copolymerization with hydrophobic polymers or blending with nanoparticles [83]. Post-loading, solvent evaporation traps RSV within the polymeric mesh.

Alternatively, this principle is adaptable in situ during hydrogel fabrication. RSV dissolved directly in the alginate precursor solution becomes physically entrapped upon ionic cross-linking, preferentially partitioning into hydrophobic niches during network formation. While operationally simple and avoiding complex synthesis, limitations include suboptimal loading efficiency due to drug precipitation during gelation and initial burst release from surface-associated or loosely bound molecules upon aqueous exposure. Mitigation strategies involve precise modulation of network density, porosity, and hydrophobicity: Enhanced cross-linking or hydrophobic moieties improve drug-matrix affinity, enabling sustained diffusion-controlled release—critical for prolonged therapeutic applications in localized cancer therapy and advanced wound dressings[90]. Passive entrapment also makes RSV release sensitive to interactions among co-loaded compounds and the surrounding polymer network. In PVA/alginate/carboxymethylcellulose films and related polymeric hydrogels, RSV maintained a progressive release profile, whereas co-incorporated curcumin disrupted matrix integrity and reduced the amount of RSV released over the same interval. Co-delivery should therefore not be assumed to preserve the release behavior of each individual agent; instead, matrix compatibility, competitive partitioning, and reciprocal effects on polymer organization require direct experimental assessment [91].

## Parameters affecting the release of resveratrol from hydrogels

The therapeutic efficacy of RSV-loaded alginate hydrogels is intrinsically linked to the controlled release kinetics of the bioactive compound from the polymeric matrix. Characterized by a stilbene scaffold comprising two aromatic rings connected by an ethenyl bridge, RSV exhibits potent therapeutic properties, including antioxidant, anti-inflammatory, and anticancer activities. However, significant physicochemical limitations persist, such as poor aqueous solubility (approximately 0.03 mg/mL in water), rapid metabolic degradation via glucuronidation and sulfation, and pronounced photosensitivity under UV exposure. These intrinsic challenges necessitate precise engineering of the release mechanism to ensure optimal bioavailability at the target site, minimizing systemic side effects [83].

The release profile of RSV from alginate hydrogel networks is governed by a complex interplay of multiple factors: the physicochemical properties of the drug, such as a molecular weight of 228.25 g/mol and log P of 3.1; the structural architecture of the hydrogel, including porosity, mesh size, and crosslinking density; and the nature of drug-polymer interactions, such as hydrogen bonding and electrostatic forces. The phenolic hydroxyl groups in RSV facilitate diverse encapsulation strategies, such as covalent conjugation through ester or amide linkages and ionic interactions with alginate’s carboxylate groups, which subsequently dictate the release behavior. For instance, covalent conjugation often leads to sustained release, whereas electrostatic interactions may result in pH-dependent kinetics [92]. Regulatory control over these parameters is essential to mitigate premature burst release, which is commonly observed in the initial phases of in vitro studies, and to achieve sustained therapeutic concentrations over extended periods. Additionally, the release kinetics exhibit responsiveness to both external and internal environmental stimuli, such as pH and temperature, which trigger hydrogel degradation, swelling, or erosion, thereby modulating drug diffusion [93]. (Fig. 1)

**Fig. 1:**
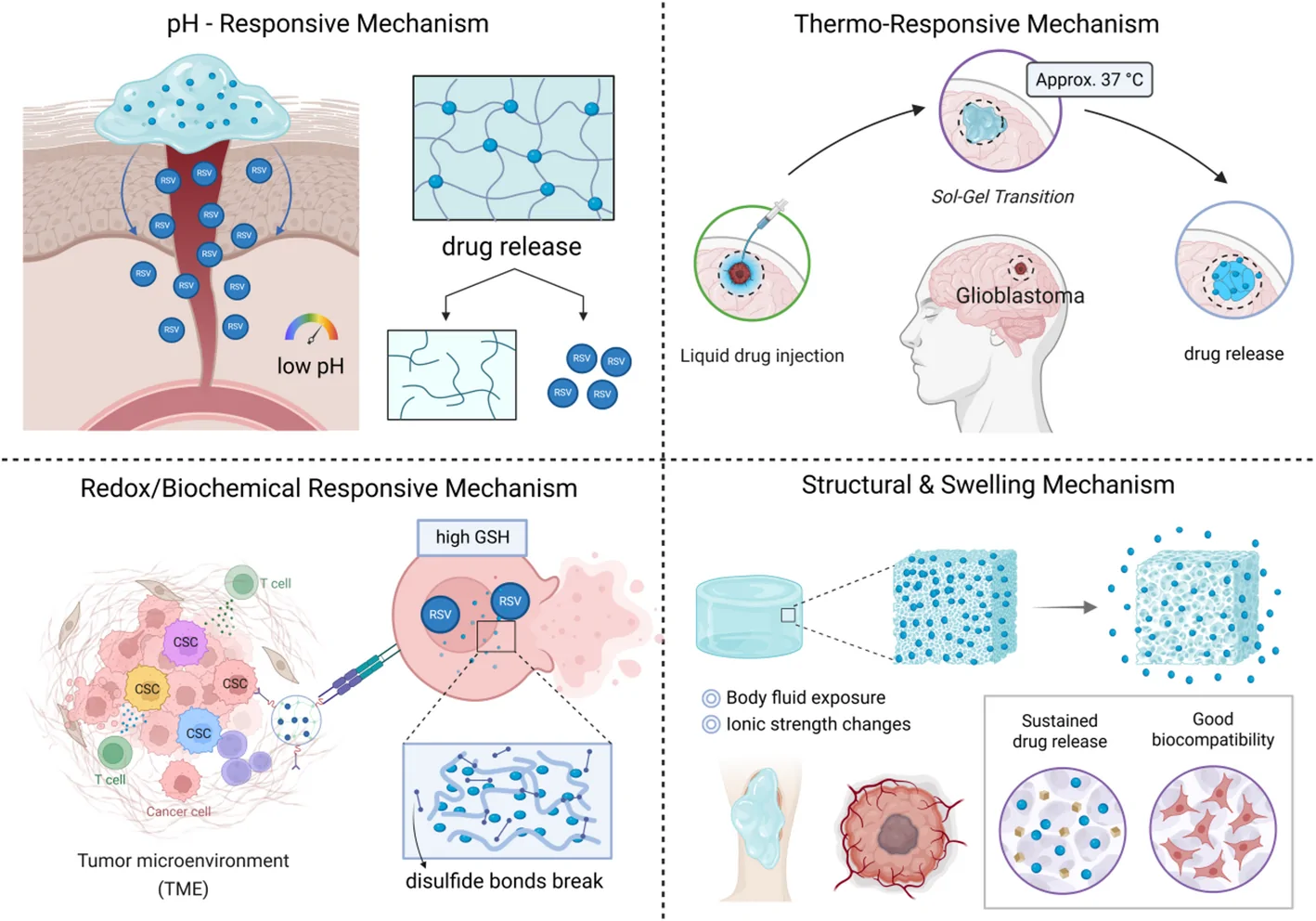
Stimuli-responsive and structural mechanisms for resveratrol release

### External stimulus

#### pH

It is well established that pH serves as a fundamental environmental parameter regulating the release behavior of therapeutic agents from stimuli-responsive hydrogel systems, particularly in alginate-based matrices. In the context of RSV delivery, the pH of the surrounding medium influences both RSV stability and the structural integrity of the alginate matrix. RSV contains phenolic hydroxyl groups that undergo appreciable deprotonation mainly under neutral-to-alkaline or basic conditions, increasing aqueous solubility at higher pH. In acidic tumor or wound microenvironments, faster release is more plausibly attributed to protonation-dependent swelling or erosion of alginate/chitosan networks and to acid-labile linker hydrolysis, rather than to strong ionization of RSV itself [81].

The design of RSV-loaded alginate hydrogels often exploits pH variations to achieve targeted drug release. For example, in covalently conjugated systems formed via ester linkages between the hydroxyl groups of RSV and the carboxylate groups of alginate, release kinetics are predominantly controlled by hydrolysis of these bonds. Such acid-sensitive linkages may hydrolyze more rapidly under mildly acidic conditions than under neutral pH, enabling site-preferential drug liberation in pathological microenvironments. This pH-responsive degradation is especially advantageous for targeted biomedical applications, such as cancer therapy and wound healing, where local pH gradients exist.

In cancer therapy, the tumor microenvironment is often mildly acidic (commonly around pH 6.5–7.0, with heterogeneity among tumor types and regions) due to altered tumor metabolism and lactate accumulation. Alginate hydrogels engineered to swell, degrade, or cleave drug linkages at lower pH values can facilitate site-specific liberation of RSV directly into tumor tissue, enhancing therapeutic efficacy while minimizing systemic toxicity [94]. Similarly, in wound healing applications, wound-bed pH is dynamic and depends on wound type, infection status, and healing stage; open wounds are frequently neutral to alkaline, whereas chronic or infected wounds often show a more alkaline microenvironment. Hydrogels responsive to these pathological pH gradients can support controlled RSV release, enabling suppression of inflammation and promotion of tissue remodeling. For instance, RSV-loaded alginate–chitosan systems with pH-modulated release have improved in vivo outcomes in infected full-thickness wounds, where controlled RSV liberation supported reduced bacterial burden, lower oxidative stress and enhanced collagen deposition [95]. Consequently, the pH-sensitivity of the delivery system must be engineered to align with the pathological conditions of the target site, leveraging alginate’s swelling and buffering properties to optimize release kinetics [96].

#### Temperature

Temperature serves as a critical external stimulus for regulating drug release kinetics and modifying the physical state of hydrogel systems, particularly in the design of injectable formulations that undergo in situ sol-gel transitions. Thermosensitive hydrogels have attracted considerable attention for RSV delivery, owing to their ability to be administered as liquids at ambient temperatures and transform into stable, gel-like depots upon exposure to physiological conditions, thereby ensuring sustained drug release with minimal invasiveness [97]. This behavior enhances encapsulation efficiency and site-specific accumulation, crucial for maintaining therapeutic efficacy in diverse biomedical applications [98].

Recent innovations have exploited thermal responsiveness to amplify the therapeutic efficacy of RSV in regenerative medicine, particularly in bone tissue engineering. For instance, an injectable thermosensitive hydrogel system based on poly(D, L-lactide)-poly(ethylene glycol)-poly(D, L-lactide) (PLEL) and loaded with RSV, combined with dexamethasone-functionalized carbonated hydroxyapatite microspheres, effectively promoted osteoporotic bone defect regeneration by facilitating osteogenic differentiation and suppressing inflammatory responses [86]. Similarly, a multifunctional hydrogel incorporating RSV-loaded thiolated F-127 micelles demonstrated sustained release profiles and enhanced osteogenesis markers, such as alkaline phosphatase and osteocalcin, alongside significant reduction in pro-inflammatory cytokines [99]. These composite systems adapt to complex defect geometries through temperature-triggered gelation, providing mechanical support while locally releasing RSV to modulate bone microenvironment dynamics [100].

Furthermore, temperature-dependent hydrogel behavior is instrumental in wound management contexts, where conformability to irregular wound beds is paramount. An injectable supramolecular gelatin hydrogel co-loaded with RSV and histatin-1 has been designed for burn wound therapy, exhibiting excellent rheological properties that allow cavity filling and antioxidant protection, thereby accelerating epithelialization and mitigating oxidative stress [90]. In the realm of cancer therapy, thermosensitive hydrogels have been adapted for intratumoral or localized delivery of RSV; a nanoemulsion-based hydrogel formulation showed enhanced cytotoxicity against breast cancer cells by enabling prolonged drug retention and controlled release kinetics [101]. Collectively, these applications highlight the versatility of temperature-responsive hydrogels in achieving high local drug concentrations and tailored therapeutic outcomes across wound healing and oncology fields [102].

### Internal stimuli

#### Porosity

Hydrogels constitute three-dimensional networks of crosslinked hydrophilic polymers dispersed in aqueous media, forming colloidal systems whose intrinsic structural attributes fundamentally regulate mass transport. Porosity—defined by pore size distribution, interconnectivity, and void fraction—is a critical determinant of diffusion kinetics for encapsulated bioactive molecules. For hydrophobic therapeutics such as RSV, the internal pore architecture governs both drug-loading capacity and diffusion pathway tortuosity, directly influencing release profiles [102].

The release mechanism typically involves drug dissolution from the polymer matrix followed by diffusion through water-filled channels. Precise modulation of network density and porosity is therefore essential to optimize RSV partitioning into hydrophobic microdomains and mitigate burst release effects associated with surface-adsorbed molecules. Engineered pore structures enable transition from rapid to sustained diffusion-controlled release, which is critical for long-term therapeutic applications such as cancer treatment and chronic wound management.

Recent studies on interpenetrating polymer network (IPN) scaffolds composed of 2-hydroxyethyl methacrylate (HEMA) and alginate demonstrate the significance of pore morphology. These scaffolds exhibit hierarchically porous structures that facilitate controlled RSV release while maintaining excellent biocompatibility and hemocompatibility [93]. Integration of nanoscale modifiers further enhances functionality: graphene nanoplatelets incorporated into sodium alginate (SA) hydrogels alter matrix porosity and drug-matrix interactions, boosting RSV retention by approximately 4.25-fold compared to free-drug formulations and enabling sustained release profiles [80]. Such modifications exploit nanomaterial-polymer synergy to optimize drug delivery kinetics without compromising hydrogel integrity.

#### Mesh size

The mesh size, defined as the average distance between consecutive cross-links within the hydrogel network, functions as a molecular sieve that critically governs the transport kinetics of encapsulated therapeutics such as RSV. The dimensions and distribution of these meshes are dynamic parameters regulated by polymer concentration, cross-linker type/density, and environmental conditions. For hydrophobic molecules like RSV, the ratio of the drug’s hydrodynamic radius to the hydrogel’s mesh size dictates the dominant release mechanism: when mesh size significantly exceeds molecular dimensions, release follows Fickian diffusion; conversely, reduced mesh size imposes steric hindrance, retarding drug efflux and enabling sustained release[103–105].

Optimization strategies for RSV delivery focus on modulating mesh size through advanced cross-linking techniques. For instance, structure-selected alginate-pectin hydrogels exploit the “egg-box” conformation to refine network density and mesh characteristics, achieving > 92% encapsulation efficiency while preventing premature leakage in the upper gastrointestinal tract. This structural control ensures targeted intestinal release, enhancing bioavailability [81].

Chemical cross-linkers further enable stimulus-responsive mesh tuning. Redox-sensitive hydrogels incorporating disulfide bridges undergo mesh expansion upon exposure to elevated glutathione (GSH) concentrations in tumor microenvironments, triggering rapid RSV release [106]. Cyclodextrin-grafted alginate networks similarly enhance drug retention through host-guest interactions, allowing controlled release kinetics [107]. Physical modification via nanofillers like graphene oxide nanoplatelets occupies interstitial spaces within alginate matrices, reducing effective mesh size and prolonging diffusion pathways. This yields significantly more controlled release profiles—sustaining drug delivery over extended periods compared to unmodified hydrogels [108].

#### Degree of crosslinking

The degree of crosslinking serves as a critical modulator of hydrogel properties, directly influencing mechanical stability, swelling capacity, and drug release kinetics in alginate-based systems. Crosslinking is achieved either through physical interactions—primarily ionic binding of divalent cations to glucuronic acid blocks—or via covalent chemical bonds. Higher crosslinking densities reduce mesh size and free volume for solvent diffusion, leading to denser networks with diminished swelling ratios. This structure retards the release of hydrophobic therapeutics like RSV, optimizing sustained delivery [109].

Physical crosslinking strategies, particularly ionotropic gelation, dominate alginate hydrogel design due to their biocompatibility and simplicity. Precision in manipulating the guluronic-to-mannuronic acid (G/M) ratio enhances the formation of stable “egg-box” junctions, thereby improving mechanical integrity and encapsulation efficiency. Such structural optimization ensures targeted intestinal release of RSV, maximizing therapeutic efficacy in gastrointestinal applications [110, 111]. Reinforcement with nanomaterials like graphene nanoplatelets further restricts polymer chain mobility and creates tortuous diffusion pathways, significantly prolonging RSV retention compared to unmodified alginate matrices. Recent studies demonstrate that graphene-enhanced systems increase drug payload capacity and exhibit pH-responsive release, advancing site-specific delivery [93].

In contrast, chemical crosslinking provides superior structural robustness and resistance to uncontrolled degradation. Innovations include injectable hydrogels leveraging “click” chemistry to form covalent networks with tunable crosslinking densities. This approach enables customized sustained-release profiles ideal for localized cancer therapy, such as in triple-negative breast cancer. Dynamic crosslinking strategies further impart stimuli-responsiveness; disulfide bridges, for example, create redox-sensitive systems. Elevated glutathione levels in tumor microenvironments trigger cleavage of these crosslinks, reducing network density and enabling accelerated, site-specific release of RSV at the tumor site [112]. Such systems demonstrate reduced off-target toxicity and enhanced therapeutic precision in preclinical models.

### Integrating hydrogel design parameters with biological outcomes

While the preceding subsections treat crosslinking density, mesh size, and porosity as independent release-modulating variables, these structural parameters ultimately converge on a single biological readout: the spatiotemporal concentration profile of bioavailable RSV at the target tissue. Rational design of RSV–alginate platforms therefore requires explicit integration of structural metrics with in vivo therapeutic endpoints.

Crosslinking density, typically manipulated through Ca²⁺ concentration (0.05–2% w/v) or the guluronic-to-mannuronic (G/M) ratio of the starting alginate, exerts a biphasic influence on therapeutic outcomes. Low crosslinking densities (e.g., < 0.5% CaCl₂) favor burst release but compromise mechanical integrity, rendering such gels unsuitable for prolonged tumor suppression. Higher crosslinking densities (> 1% CaCl₂ or G/M > 1.5) extend release duration to 72 h or beyond and have been empirically correlated with superior wound contraction—such as achieving over 80% closure within 14 days in rodent models—and sustained antitumor effects. Importantly, this correlation is not monotonic: excessive crosslinking creates a densely packed matrix that impairs cellular infiltration and paracrine signaling, paradoxically blunting the regenerative response despite improved drug retention. The optimal window therefore depends on the therapeutic intent—maximal cellular infiltration (wound healing) vs. maximal drug residence (intratumoral depot).

Mesh size exerts the most direct control over RSV diffusion kinetics. When the ratio of the RSV hydrodynamic radius (∼0.48 nm) to mesh size exceeds ∼0.1, release transitions from Fickian to sterically hindered, shifting the drug concentration profile from a rapid-decay to a sustained-plateau regime. This transition carries mechanistically distinct biological consequences: sustained plateaus favor chronic inflammatory modulation, such as persistent NF-κB suppression and fibroblast recruitment in chronic wounds, whereas burst kinetics are more appropriate for acute oncological contexts requiring supratherapeutic peak concentrations to trigger apoptosis.

Porosity and pore interconnectivity additionally govern cellular infiltration and ECM remodeling. Pore sizes in the 100–300 μm range have been consistently associated with improved vascular ingrowth and optimal fibroblast/keratinocyte migration in wound beds, whereas sub-100 μm porosity is preferable for intratumoral depot injections where cellular infiltration is undesirable. Reports of pore-size-dependent re-epithelialization rates (∼2.3-fold acceleration in 200 μm-pore systems versus 50 μm controls) underscore that identical alginate chemistry can yield divergent therapeutic outcomes depending solely on architectural tuning.

Taken together, the literature supports three practical design heuristics: (i) for chronic wound management, favor a moderate crosslinking density of 0.5–1% CaCl₂, large pores of ≥ 150 μm, and release durations of 48–72 h; (ii) for localized oncological depot delivery, favor higher crosslinking density (> 1% CaCl₂), smaller mesh sizes to ensure plateau kinetics, and stimulus-responsive linkers such as GSH-cleavable disulfides or acid-labile esters; and (iii) where inter-batch reproducibility is critical, the G/M ratio must be specified alongside polymer concentration, since variability in alginate source material is a dominant driver of inter-study discrepancies.

Several quantitative anchors warrant explicit referencing. The biphasic crosslinking-density–release relationship has been directly demonstrated for low-molecular-weight aromatic acids in calcium-alginate matrices, where the apparent diffusion coefficient decreases monotonically with crosslinker concentration up to a saturation regime [113]; the typical operating window for spray-dried alginate microparticles spans 0.05–0.5% CaCl₂ with prolonged drug release demonstrated up to 12 h [114]. The hydrodynamic radius of RSV (MW 228 Da) is most reliably estimated from the Stokes–Einstein relation at ∼0.4–0.6 nm in aqueous buffer at 25 °C; this estimate is consistent with the ∼0.48 nm value adopted in this Section but should be experimentally verified by dynamic light scattering or pulsed-field-gradient NMR for any specific formulation. Pore-size dependence of cellular response in wound beds is well established: small pores (1–2 μm) favor epidermal attachment, intermediate pores (40–400 μm) support vascularization, and pores > 300 μm promote direct osteogenesis and angiogenesis [115], which collectively support the 100–300 μm operating window recommended above. Recent advances in digital-light-processing bioprinting now permit deterministic fabrication of gradient pore architectures within hydrogel scaffolds, opening a route to spatially programmed cellular responses.

## Application of resveratrol-loaded alginate hydrogels

Alginate-based hydrogel dressings significantly accelerate wound closure, enhance re-epithelialization, and promote extracellular matrix (ECM) remodeling compared to conventional treatments [116, 117]. Additionally, incorporating bioactive components such as graphene nanoplatelets or pectin into alginate hydrogels creates an efficient platform for smart drug delivery systems, substantially improving drug retention and enabling controlled release for both oncology and regenerative medicine applications.

### Wound healing

Alginate hydrogels represent a promising platform for wound healing applications owing to their inherent biocompatibility, porous architecture, high water retention capacity, and favorable permeability for both aqueous and gaseous exchange [118]. This section examines the efficacy of RSV-loaded alginate hydrogels and their modified copolymeric networks in advancing wound regeneration. (Fig. 2)

**Fig. 2:**
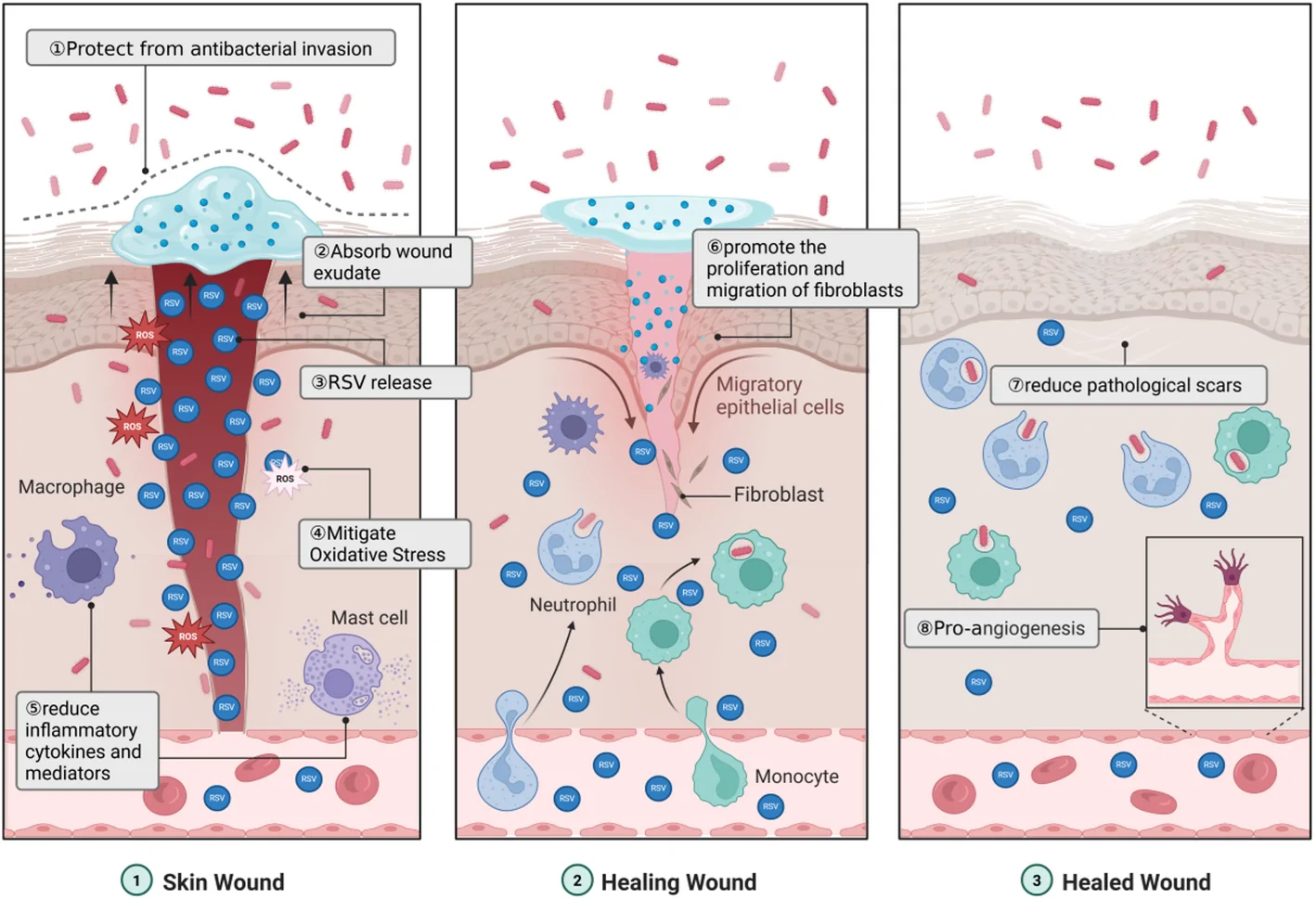
Schematic illustration of the multifaceted mechanisms of resveratrol-loaded alginate hydrogels in promoting wound healing

#### Alginate-based and chitosan-containing systems for wound healing

Direct evidence for RSV-loaded alginate wound dressings is provided by an ionically cross-linked sodium alginate film developed by Lu et al. The film combined high drug content, sustained local RSV release, and improved mechanical stability; in an animal burn model, it accelerated tissue regeneration and achieved 86.09% re-epithelialization by day 14 [119]. Complementary formulation-level evidence was reported by Comotto et al., who separately incorporated t-resveratrol or curcumin into Ca²⁺-cross-linked alginate/glycerol patches. The alginate platform maintained favorable mechanical and oxygen-permeability characteristics, while the t-resveratrol formulation exhibited an initial burst followed by diffusion-dominated release over 72 h, remained compatible with human keratinocytes at concentrations up to 300 µg mL⁻¹, and promoted scratch closure at 300 µg mL⁻¹ [120]. Collectively, these studies support the use of alginate as both a hydrated wound interface and a local RSV depot. Nevertheless, direct in vivo therapeutic evidence remains largely confined to the animal burn model reported in study.

Adding chitosan expands this platform from sustained delivery to staged management of infected wounds. Wang et al. fabricated RSV-loaded alginate-chitosan (RSV@ALG-CS) core-shell microcapsules by microfluidic electrospray, with RSV in the alginate core and an imine-cross-linked chitosan shell. The shell reduced premature RSV leakage and degraded under acidic conditions, coupling antimicrobial action with subsequent RSV-mediated ROS scavenging. In Staphylococcus aureus-infected wounds, the system reduced bacterial burden, oxidative stress, and inflammation while promoting collagen deposition and angiogenesis [95]. Thus, the alginate core provides encapsulation and spatial control, whereas the chitosan shell contributes barrier stability and infection-responsive release.

Network architecture provides a second design lever. In HEMA/alginate interpenetrating polymer network (IPN) scaffolds, cross-linker type and concentration altered interconnected porosity, swelling, mechanical properties, biocompatibility, and controlled RSV release [83]. Because this study assessed release behavior and biocompatibility rather than wound closure, it should be interpreted as formulation-level support for tuning alginate networks, rather than direct evidence of therapeutic wound healing.

Related hydrogel systems suggest two transferable approaches for improving RSV solubility and stimulus-responsive release. Radeva et al. incorporated hydroxypropyl-β-cyclodextrin-complexed RSV into a Pluronic F-127 hydrogel; the formulation improved aqueous handling and showed a favorable fibroblast scratch-assay trend and antimicrobial activity, but contained no alginate [121]. An alginate-free chitosan double-network hydrogel subsequently used oxidized β-cyclodextrin as both an RSV host and a cross-linker, enabling pH-responsive release and improving healing in a murine chronic infected-wound model [122]. These studies are therefore design analogues rather than direct RSV-alginate evidence. Overall, the field has progressed from simple alginate films to alginate-chitosan core-shell constructs, while cyclodextrin complexation and dynamic cross-linking offer rational next steps for multifunctional RSV-alginate wound dressings.

#### Nanocomposites and scaffolds

Efficient wound management necessitates accelerated re-epithelialization of the epidermal layer and enhanced collagen deposition. To this end, Ma et al. designed a bi-layered wound dressing that mimics the epidermal–dermal architecture of skin, comprising an upper resveratrol-loaded poly(lactic-co-glycolic acid) (Res-PLGA) electrospun nanofiber mat and a lower alginate di-aldehyde (ADA)–gelatin (GEL) crosslinked hydrogel. The construct showed good hemostatic capacity and appropriate swelling behaviour, and both HaCaT keratinocytes and human embryonic skin fibroblasts grew well within their respective layers. In a Sprague–Dawley rat full-thickness wound model, the resveratrol-loaded bi-layered scaffold accelerated wound closure relative to the Res-PLGA mat or the ADA–GEL hydrogel alone, demonstrating that pairing a nanofibrous drug reservoir with an alginate-based hydrogel layer can integrate sustained RSV delivery with a hydrated, cell-supportive wound interface [123]. The injectable hydrogel exhibited diffusion-controlled RSV release in initial stages, transitioning to erosion-dominated kinetics in later phases. In vivo studies confirmed its efficacy in dorsal wound healing in rats, demonstrating significant improvements in re-epithelialization and collagen deposition within wounded tissues [121].

Beyond such layered constructs, calcium alginate matrices can also function as a shared depot for RSV-loaded nanoparticles and regenerative cells. The simultaneous incorporation of RSV nanocarriers and adipose-derived stem cells couples sustained antioxidant protection with cell-mediated paracrine repair, thereby supporting re-epithelialization, collagen organization, vascular remodeling, and recovery of photoaged skin. This hybrid strategy extends RSV–alginate systems beyond passive drug release toward coordinated pharmacological and cellular therapy [124].

Chondroitin sulfate has emerged as a potent candidate for wound repair. Shah et al. synthesized injectable hydrogels combining Chondroitin sulfate and SA via solvent casting, co-loaded with RSV and stabilized with Pluronic PF-127 [125]. This formulation synergistically promoted re-epithelialization, angiogenesis, and collagen aggregation within the wound microenvironment. Controlled release kinetics of encapsulated drugs were observed, alongside superior biocompatibility validated through in vitro studies on 3T3-L1 fibroblast cells and in vivo toxicity assessments in subcutaneous animal models [126, 127].

Further advancing this approach, a hybrid nano-scale scaffold integrating collagen-SA hydrogels with RSV-CS nanoparticles was designed. These scaffolds displayed enhanced biocompatibility, elevated water uptake capacity, improved solubility/stability, and optimized RSV release profiles [128]. In vivo administration significantly accelerated wound contraction rates. Additionally, treated animals exhibited complete epithelialization with extensive granulation tissue formation, confirming the scaffold’s regenerative potential.

Further functional separation can be achieved through multilayer architectures rather than incorporating all therapeutic components into a single homogeneous matrix. A dual-layered construct combining an electrospun PVA–alginate layer containing green-synthesized silver nanoparticles with a lyophilized alginate hydrogel containing RSV-loaded ZIF-8 provides spatially distinct antibacterial and antioxidant compartments. This biomimetic organization allows the outer layer to strengthen antimicrobial protection and structural support, while the hydrogel compartment regulates RSV retention and release within the wound-facing region [129].

#### Multi-drug delivery and emulsion systems

Numerous polysaccharides are utilized either individually or in combinations for effective wound management, among which carrageenan is a prominent example [130]. Considering this, Postolovic et al. developed carrageenan-SA-poloxamer 407 (P407)-based hydrogels integrated into films to facilitate the dual drug delivery of diclofenac (DF) and RSV for wound healing applications. Three distinct hybrid films were fabricated: a DF-loaded film, a RSV-loaded film, and a dual drug-loaded (DF/RSV) film. Enhanced encapsulation efficiency (%EE) for both agents enabled the observation of a synergistic effect, and the co-loaded hybrid films further demonstrated antibacterial activities against Bacillus subtilis and Staphylococcus aureus, functioning as multifunctional therapeutic agents for inflammation treatment, pain mitigation, and accelerated wound healing progression [131].

Hydrogels remain a cornerstone in wound healing research. In a separate study, researchers engineered Pickering emulsion-hydrogel nanocomplex systems composed of RSV, SA, and carboxymethyl chitosan (CMCS), stabilized with P407 [132]. The influence of ionic concentrations and temperature on the morphology and average diameter of these synthesized emulsions was systematically investigated, with the Pickering structure facilitating controlled drug release. These nanocomplex hydrogels exhibited potent antibacterial effects against Escherichia coli and Staphylococcus aureus, alongside enhanced wound healing efficacy, as evidenced by elevated levels of Ki67 and CD31 expression, upregulated TGF-β3 mRNA, and reduced α-SMA and TGF-β1 mRNA transcripts.

#### Advanced functionalized systems and nanocarriers

Zhang et al. engineered a hybrid hydrogel system incorporating RSV, epigallocatechin gallate (EGCG), hyaluronic acid (HA), SA, and polylysine (PLL) for the effective management of irradiation-induced skin injury. Five distinct hydrogel formulations were evaluated for hemocompatibility, adhesion to fresh blood, and hemolysis ratios. The hybrid hydrogels demonstrated pronounced anti-biofouling effects by effectively inhibiting protein and bacterial adhesion while evading immune responses [133, 134]. Furthermore, the system exhibited sustained release kinetics for both RSV and EGCG, significantly accelerating skin wound healing through enhanced anti-inflammatory and antioxidant effects and promotion of angiogenesis [135].

In a separate study, a chitosan (CS)–polyvinyl alcohol (PVA)–SA hydrogel loaded with RSV nanoparticles and functionalized with mesoporous Ag₂O/SiO₂ was developed for transdermal applications. The impact of varying Ag₂O/SiO₂ concentrations revealed that increased nanoparticle content markedly improved mechanical strength and wound healing efficacy. The hybrid formulation exhibited robust antibacterial activity against Staphylococcus epidermidis, Acinetobacter baumannii, and Proteus mirabilis, validating its potential for infected wound management [136].

Nguyen et al. designed a topical RSV-loaded hydrogel conjugated with calcium alginate-based nanocarriers (CSNs) using a nanoemulsification-polymer crosslinking technique. The CSNs displayed a hydrodynamic size distribution of ∼200 nm, a surface charge of ∼−30 mV, and excellent biocompatibility. RSV-loaded CSNs achieved high encapsulation efficiency (∼95%) and demonstrated potent antioxidant effects. Human skin studies confirmed significant RSV accumulation in the upper epidermal layers, highlighting the utility of these hydrogel-based nanocarriers for wound healing and cosmeceutical applications [137, 138].

The development of efficacious hydrogel systems for wound management necessitates optimization of multiple factors, including drug encapsulation efficiency, release kinetics, degradation patterns, hydrogel degradability, biocompatibility, and mechanical/physicochemical properties. RSV-loaded alginate hydrogels offer a cost-effective platform that can be functionally tailored for targeted applications [139]. Their intrinsic biocompatibility enables further enhancement through the integration of multifunctional architectures, such as antioxidant or angiogenic moieties, to optimize therapeutic outcomes [140].

### Cancer therapy

Alginate-based hydrogels exhibit considerable potential in biomedical applications, particularly in oncology, owing to their high water retention capacity, non-toxicity, biocompatibility, and biodegradability. These properties enable their utility as efficient carriers for chemotherapeutic agents and biomolecules to target sites. Notably, drug release from alginate hydrogel networks is primarily governed by diffusion-mediated, swelling-mediated, chemically mediated, and environment-responsive mechanisms [141]. This section synthesizes recent advances in RSV-loaded alginate hydrogels for anticancer applications.

#### Co-delivery and targeted hydrogel systems

Co-delivery and targeted hydrogel systems represent an important application-oriented strategy for enhancing RSV-based anticancer therapy. In this section, “targeted” mainly refers to improved local retention and tumor-site release, rather than fully validated receptor-mediated targeting. Because the physicochemical and pharmacokinetic limitations of RSV have been discussed above, the following discussion focuses on representative cancer-oriented formulations and their therapeutic outcomes.

One representative strategy is the co-delivery of RSV with another bioactive polyphenol. Saralkar and Dash developed calcium alginate nanoparticles co-encapsulating curcumin and RSV and evaluated their activity against DU145 prostate cancer cells [142]. This formulation improved the aqueous handling of both hydrophobic compounds and provided an alginate-based carrier suitable for combination therapy. More recently, Siripruekpong et al. reported an in situ gelling, raft-forming gastroretentive formulation for the simultaneous delivery of curcumin and RSV. The optimized sodium alginate-based system showed sustained release in simulated gastric fluid and enhanced cytotoxicity against AGS gastric adenocarcinoma cells compared with free compounds, supporting the use of alginate-containing formulations for localized gastrointestinal cancer therapy [143].

Injectable and locally retained hydrogel systems provide another route for improving tumor-confined RSV therapy. Melo et al. developed chitosan-based injectable in situ-forming hydrogels containing RSV and DOPA-rGO for breast cancer chemo-photothermal therapy. Although this system is not alginate-based, it is mechanistically relevant to polysaccharide hydrogel design because it demonstrates how RSV can be retained locally and combined with a physical therapeutic modality. Under NIR irradiation, the RSV/DOPA-rGO hydrogel reduced breast cancer cell viability to 31%, whereas the corresponding single-modality treatments showed weaker effects [144]. This example should therefore be used as supportive evidence for multimodal RSV–hydrogel therapy, while the alginate-specific discussion should remain centered on alginate-containing co-delivery systems.

Overall, co-delivery and targeted hydrogel systems extend RSV-based cancer therapy beyond simple encapsulation by combining local retention with complementary anticancer agents or modalities. For this subsection, the strongest evidence supports curcumin–RSV co-delivery in alginate-containing systems and related polysaccharide hydrogel platforms for chemo-photothermal treatment. Claims regarding active receptor-mediated targeting should remain cautious until directly validated in RSV-loaded alginate hydrogels. This focused discussion avoids overlap with the following section, where 3D tumor models and graphene oxide-containing alginate composites are considered in greater detail.

#### 3D Tumor models for RSV evaluation

In alignment with analogous objectives for cancer treatment, Wang et al. developed an in vitro 3D co-cultured tumor-vascular barrier (3D-TVB) model employing alginate hydrogel beads and a Transwell system. Initially, the alginate hydrogel beads were encapsulated with prostate cancer (PC-3) cells and fibroblast (NIH/3T3) cells, which were subsequently cultured in the lower compartment of the Transwell chamber. Concurrently, human umbilical vein endothelial cells (HUVECs) were cultured on the permeable membrane within the upper cavity to establish a vascular barrier. The evaluation of RSV response in this 3D-TVB model revealed significantly enhanced anticancer activity compared to the 2D-TVB counterpart, underscoring the model’s superiority in mimicking the tumor microenvironment [145]. This study highlighted the utility of microfluidic approaches for recapitulating complex cancer microenvironments, thereby facilitating real-time and accurate assessment of therapeutic processes [2].

#### Microparticulate and pH‑sensitive systems

In addition to conventional hydrogel particles, alginate-functionalized metallic nanocarriers provide a compact platform for combining RSV delivery with mitochondria-associated oxidative stress. Sodium alginate-coated silver nanoparticles can improve RSV dispersion and cellular internalization while promoting mitochondrial ROS accumulation and caspase-associated apoptosis in MCF-7 breast cancer cells. The alginate coating contributes colloidal stabilization and drug retention, whereas the metallic core provides an additional source of intracellular oxidative stress that may amplify the pro-apoptotic activity of RSV [146].

Replacing the silver core with gold enables a more explicit pH-responsive delivery design. Sodium alginate-functionalized gold nanoparticles loaded with trans-resveratrol exhibited a particle size of approximately 24 nm and released the payload more readily under acidic than physiological conditions, consistent with preferential drug liberation in an acidified tumor microenvironment. The formulation reduced MCF-7 cell viability to approximately 29% at 12 nM while increasing mitochondrial oxidative stress, indicating that pH-sensitive release and intracellular ROS generation can be integrated within the same alginate-functionalized nanocarrier. Nevertheless, these findings remain limited to in vitro breast cancer models and require pharmacokinetic and in vivo validation [147].

Further exploring the capabilities of RSV-loaded alginate hydrogel systems, Wezgowiec et al. developed micro- and macro-particles encapsulated in SA-based hydrogels and evaluated their efficacy against human colon cancer cells (LoVo). The study also assessed synergistic effects of polymeric coatings (chitosan and gelatin) on RSV-loaded micro/macroparticles, revealing that microparticles exhibited lower drug encapsulation efficiency but more sustained release kinetics compared to macroparticles [148]. Negligible cytotoxicity was observed for empty particles, whereas RSV-loaded systems significantly reduced LoVo cell viability, with gelatin-coated or uncoated microparticles identified as the most potent carriers [149].

In a complementary approach, pH-sensitive interpenetrating polymeric network (IPN) hydrogels were fabricated using SA-g-poly(N-acryloyl-L-phenylalanine), ethylene glycol vinyl ether, and hydroxyethyl acrylate (HEA) via free-radical polymerization [150]. These in-situ-RSV-loaded hydrogels demonstrated 75% encapsulation efficiency and superior antiproliferative effects against MCF-7 breast cancer and HepG2 hepatic carcinoma cells relative to free RSV [111]. Histopathological analysis confirmed reduced tissue toxicity compared to controls, while in vivo studies further validated their tumor-suppressive efficacy [2].

#### Hydrogels as ECM mimics

Hydrogels constitute an optimal platform for targeted drug delivery to cancerous tissues, owing to their inherent biocompatibility, protective encapsulation capabilities, and facile handling characteristics. Beyond their role as drug carriers, hydrogels can be engineered to emulate the ECM, thereby establishing a three-dimensional (3D) microenvironment that supports cell culture and tissue engineering applications. The porosity and interconnectivity of the hydrogel matrix critically influence cellular behaviors, including adhesion, proliferation, migration, and differentiation, through modulation of mechanical and biochemical cues [151]. These properties are pivotal for enhancing therapeutic efficacy in cancer treatment, as they facilitate tumor-specific drug release and cellular responses [152]. Moreover, recent advancements in RSV-loaded alginate hydrogels demonstrate their multifunctionality across cancer therapy and wound healing, leveraging alginate’s tunable biodegradability and RSV’s bioactivity to optimize outcomes [153]. To complement the qualitative summary above, Table 2 consolidates the key quantitative performance metrics—particle size, encapsulation efficiency, release duration, and principal therapeutic outcomes—reported for representative RSV–alginate hydrogel systems. ECM-mimetic models can also be used to determine whether an RSV delivery system merely releases its payload or actively overcomes stromal barriers. Incorporating RSV together with hyaluronidase into polydopamine-based nanomedicine enables simultaneous pharmacological treatment and enzymatic degradation of hyaluronic acid-rich matrices. In a pancreatic tumor-sphere model enriched with hyaluronic acid, this strategy improved access to the cellular compartment and suppressed the invasive phenotype, illustrating how matrix remodeling can complement RSV-mediated tumor inhibition [154]. Although this formulation is not alginate-based, the same principle could be translated to alginate depots that locally co-deliver RSV and ECM-modulating enzymes.

**Table 1:** Representative RSV-loaded alginate-containing delivery systems for wound healing, anticancer evaluation, and site-specific drug delivery

System composition	Formation/crosslinking method	Crosslinking or stabilizing agent	Payload	Application/study model	Key verified outcomes	Ref.
RSV-loaded SA hydrogel film	Solution casting followed by ionic crosslinking	CaCl₂	RSV	Burn wound healing in rats	Sustained RSV release from the crosslinked film; accelerated wound closure and re-epithelialization	[119]
Alginate hydrogel dressing	Ca²⁺-diffusion ionic crosslinking using an agarose/CaCl₂ mold	CaCl₂; glycerol	t-RSV or curcumin, evaluated separately	In vitro wound-dressing evaluation	Maintained mechanical properties and oxygen permeability; showed antibacterial activity, keratinocyte compatibility, and in vitro scratch-closure activity	[120]
ALG–CS core–shell microcapsules	Microfluidic electrospray followed by core–shell microcapsule formation	Alginate core; imine-crosslinked CS shell	RSV	Infected full-thickness wounds	Controlled RSV release; reduced bacterial burden and oxidative stress; promoted collagen deposition and angiogenesis	[95]
SA/GNP composite beads	Ionic gelation of alginate beads with nanocomposite reinforcement	CaCl₂; graphene nanoplatelets	RSV	pH-responsive controlled drug delivery	GNP incorporation improved release control while retaining normal-cell biocompatibility under the tested conditions	[80]
Curcumin/RSV calcium alginate nanoparticles	Emulsification–crosslinking nanoparticle preparation	Ca²⁺-mediated alginate gelation	Curcumin and RSV	In vitro evaluation in DU145 prostate cancer cells	Enabled co-encapsulation and differential release of the two polyphenols; drug-loaded nanoparticles reduced DU145 cell survival, although RSV cellular uptake remained limited	[142]
Curcumin/RSV SA raft-forming in situ gel	Curcumin/RSV solid dispersion incorporated into an in situ gelling gastroretentive formulation	CaCO₃-derived Ca²⁺; Eudragit EPO solid dispersion	Curcumin and RSV	Gastroretentive delivery; in vitro AGS gastric adenocarcinoma evaluation	Rapid raft formation and sustained release in simulated gastric fluid; enhanced cytotoxicity against AGS cells compared with the unformulated compounds	[143]

**Table 2:** Representative quantitative performance metrics for resveratrol-loaded alginate-containing delivery systems

System composition	Particle/feature size or dosage form	Encapsulation efficiency/loading	Release profile/duration	Principal experimentally verified outcome	Ref.
RSV-loaded SA hydrogel film	Crosslinked film; thickness approximately 0.053 ± 0.005 mm	NR	Optimized crosslinked formulation released approximately 20.49 ± 0.28% of RSV over 24 h	Accelerated burn-wound closure and re-epithelialization in rats during a 14-day study	[119]
Alginate hydrogel dressing containing t-RSV or curcumin	Hydrogel patch; approximately 1 mm thick; drug concentration 150–300 µg/mL	NR	Burst release followed by gradual diffusion-controlled release; t-RSV release reached 55% and 42% at 72 h for the 150 and 300 µg/mL formulations, respectively	Supported keratinocyte viability and demonstrated antibacterial and in vitro scratch-closure activity; no in vivo evaluation was performed	[120]
RSV@ALG–CS core–shell microcapsules	ALG microspheres: approximately 300 μm; ALG–CS microcapsules: approximately 244 μm after shell crosslinking-induced shrinkage	EE: 69.22 ± 3.15%; drug loading: 0.653 ± 0.021%	Approximately 60% RSV release after 48 h; pH-modulated sustained release	In infected full-thickness wounds, residual wound area decreased to 12.57% on day 9; collagen deposition reached 69.7 ± 3.4% on day 9 and 80.24 ± 2.3% on day 14	[95]
SA/GNP composite beads	Alginate/GNP composite beads; size NR	NR	pH-responsive RSV release; GNP reinforcement improved release control	Maintained normal-human-cell biocompatibility under the tested conditions	[80]
Curcumin/RSV calcium alginate nanoparticles	12.53 ± 1.06 nm in nanosuspension; 60.23 ± 15 nm after freeze-drying	Curcumin EE: 49.3 ± 4.3%; RSV EE: 70.99 ± 6.1%	At 24 h, curcumin and RSV release reached 16.3 ± 3.1% and 87.6 ± 7.9%, respectively	Drug-loaded nanoparticles reduced DU145 cell survival relative to blank nanoparticles; RSV cellular uptake remained poor	[142]
Curcumin/RSV SA raft-forming in situ gel	Gastroretentive raft-forming in situ gel	NR	Floating lag time < 60 s; >80% of curcumin and RSV released within 8 h in simulated gastric fluid	Optimized medium-viscosity SA formulation showed greater cytotoxicity against AGS cells than unformulated curcumin and RSV	[143]
ALG/LMP oral hydrogel carrier for RSV	Alginate/low-methoxyl pectin oral hydrogel carrier; size NR	EE: 92.04 ± 0.32%; loading: 6.41 ± 0.022 mg/g	94.71% of RSV was transported to the intestine within 2 h; release occurred mainly in the small intestine and colon	Protected RSV under simulated gastric conditions and enabled intestine-targeted release	[81]

Note: NR = not reported or not extractable from the cited source. Values were retained only when directly traceable to the cited original study

## Clinical translation: barriers and opportunities

Despite an abundance of promising preclinical data, RSV-loaded alginate hydrogels remain at the preclinical stage, although limited human evidence from non-alginate or incompletely characterized topical RSV-gel formulations provides an early clinical proof of concept. A dispassionate assessment of the translational landscape is therefore essential for guiding future research priorities, and for contextualizing the efficacy metrics summarized in Tables 1 and 2.

### Scalability and manufacturing reproducibility

Bench-scale RSV–alginate systems are commonly prepared by batch ionic gelation with manually controlled addition of CaCl₂. The wide encapsulation-efficiency range reported across the literature reflects differences in polymer grade, drug-to-polymer ratio, mixing, crosslinking, washing, and analytical methods and should not be interpreted as direct evidence of batch-to-batch variability. True reproducibility must instead be demonstrated through replicate manufacturing runs performed with predefined critical material attributes and process parameters. Inter-grade and inter-batch differences in sodium alginate have been linked to variations in molecular-weight distribution, rheological behavior, swelling, erosion, and drug release, indicating that incoming-material specifications should include molecular weight, viscosity profile, G/M ratio, and gelation performance rather than relying only on the nominal grade [155, 156]. For parenteral or implantable formulations, these functional attributes should be complemented by product-specific acceptance criteria for bioburden, endotoxin, elemental impurities, and other source-derived contaminants.

Sterilization should be developed as an integral component of formulation design because the alginate matrix and RSV may respond differently to the same process. Exposure of commercial alginate samples to γ-irradiation at 15 kGy resulted in an average molecular-weight reduction of at least 50%, with a maximum reduction of approximately 84% among the tested grades, accompanied by marked losses in viscosity and gelation-related performance [157]. By contrast, electron-beam irradiation of unformulated RSV at 25 kGy produced no detectable degradation products or trans-to-cis conversion by HPLC and HPLC–MS, although assay-dependent changes in antioxidant activity were observed [158]. These findings indicate that the radiation stability of the isolated active compound cannot be extrapolated to an RSV-loaded alginate network. Product-specific sterilization studies should therefore evaluate alginate molecular integrity, RSV potency and related substances, rheological and mechanical properties, release kinetics, sterility assurance, and packaging compatibility before and after processing.

Low-temperature processing may help protect RSV, but the function of each technology should be described accurately. Supercritical-CO2 processing has been used to prepare RSV-loaded alginate aerogels and may be useful for loading or drying thermolabile formulations [159]; it should not be presented as a sterilization method unless a product-specific process has demonstrated the required sterility assurance level. Where terminal sterilization cannot preserve the predefined quality profile, aseptic manufacture of sterile-filtered precursor solutions or a lyophilized precursor kit for point-of-use reconstitution may be more appropriate, provided that mixing consistency, dose uniformity, and usability are validated.

### Alginate source variability and regulatory considerations

Alginate is a naturally sourced polysaccharide whose G/M ratio, molecular-weight distribution, viscosity, and functional performance can vary with biological source and manufacturing process. Ultrapure, well-characterized alginate grades, including PRONOVA materials, provide useful starting points for parenteral or implantable development because their composition and impurity profiles are more tightly controlled than those of conventional commercial alginates. Nevertheless, suitability remains product-specific. Specifications should define identity, molecular-weight distribution, G/M ratio, viscosity, and gelation performance, together with route-appropriate limits for endotoxin, bioburden, elemental impurities, and other source-derived contaminants. Where an alginate supplier maintains an appropriate master file, a right of reference may facilitate review of proprietary manufacturing and quality information, but no individual commercial grade should be described as a universal regulatory requirement.

An RSV-loaded hydrogel may meet the definition of a drug-device combination product under 21 CFR 3.2(e), but its lead FDA center and principal marketing pathway would be determined by the product’s primary mode of action. If the pharmacological activity of RSV provides the most important therapeutic action, CDER would generally be expected to serve as the lead center, with CDRH consultation where the hydrogel or delivery component raises device-specific questions. When classification or center assignment remains uncertain, early Pre-RFD or formal RFD engagement with the Office of Combination Products would be appropriate.

Regulatory planning should distinguish FDA performance goals from observed median review times. MDUFA V establishes submission-specific performance targets, whereas total elapsed development and review time also depends on application type, review cycles, major deficiencies, sponsor-response time, manufacturing readiness, and the need for inter-center consultation. A universal 6-18-month delay should therefore not be attributed to combination-product status. A credible timeline for an RSV-alginate formulation should be constructed only after its primary mode of action, lead center, investigational application, and anticipated marketing pathway have been clarified.

Within the European Union, an acellular RSV-alginate hydrogel would not ordinarily qualify as an advanced therapy medicinal product unless it incorporated gene-, cell-, or tissue-engineered components meeting the relevant legal definitions. Its regulatory status as a medicinal product, medical device, or integral drug-device combination should instead be assessed case by case according to its composition, intended purpose, and principal intended action, with quality and clinical documentation aligned to the resulting classification.

These classification issues help explain why many injectable hydrogels that have reached clinical evaluation or the market are regulated as devices, whereas drug-containing depots remain less common [160]. Incorporation of RSV may introduce requirements for dose justification, local and systemic pharmacokinetics, drug-product stability, related-substance control, and controlled clinical evidence in addition to the performance testing expected for the hydrogel component. First-in-human development should therefore combine an appropriately justified safety programme with adequately powered efficacy endpoints and a clinically meaningful comparator.

### Long-term safety and residual impurity management

Long-term safety assessment should begin with a chemically defined leachables profile rather than a universal numerical limit for EDC/NHS. Residual coupling reagents and reaction by-products should be quantified using validated analytical methods after washing and sterilization, and their allowable levels should be justified from estimated patient exposure and toxicological evaluation under ISO 10993-18 and ISO 10993-17. Recent EDC/NHS-crosslinked collagen and alginate systems further demonstrate that coupling conditions influence network structure, swelling, degradation, and biological performance, making reaction stoichiometry, washing efficiency, residual analysis, and batch consistency critical quality attributes [161, 162].

The duration and scope of biological evaluation should be derived from the intended route, tissue-contact category and duration, degradation behavior, and potential local and systemic exposure rather than from a universal 90-day requirement. Long-term implanted depots may require implantation, systemic-toxicity, histopathology, toxicokinetic, degradation-product, and RSV-metabolite assessments, whereas topical wound dressings should be evaluated using endpoints appropriate to breached or compromised skin. Where the hydrogel or a constituent part is evaluated as a medical device, the resulting programme should be constructed within a risk-based framework incorporating the current editions of ISO 10993-1, ISO 10993-6, ISO 10993-10, ISO 10993-11, ISO 10993-17, ISO 10993-18, and ISO 10993-23, with study duration justified by the expected clinical residence time.

For natural-polymer hydrogels, source-derived contaminants, leachables, sterilization-induced changes, and degradation products should be integrated into a single biological-evaluation plan rather than assessed as independent endpoints. LC-MS/MS profiling, validated RSV potency and related-substance assays, sensitization and irritation testing, local implantation or wound-tolerance studies, systemic exposure assessment, and haemocompatibility testing where blood contact is expected should be selected according to a documented risk analysis. This approach converts long-term toxicity from a generic data gap into a product-specific programme linked to material composition, route, dose, and residence time.

### Heterogeneity of preclinical evidence and conflicting findings

A recurring methodological concern is the lack of standardization across preclinical studies. Rodent wound models vary in wound diameter, depth, diabetic status, splinting, treatment frequency, comparator selection, and endpoints such as planimetry, histology, collagen deposition, angiogenesis, and inflammatory markers. Tumor studies differ in cell line, implantation site, immune status, hydrogel dose, RSV loading, and treatment schedule. This heterogeneity limits direct cross-study comparison and prevents apparent differences in efficacy from being attributed confidently to hydrogel composition alone. Preclinical reports should therefore provide complete formulation composition, alginate molecular characteristics, crosslinking conditions, loading and release methods, sterilization status, replicate-batch data, prespecified endpoints, and blinded outcome assessment.

Mechanistic interpretation also requires caution. Apparent direct activation of SIRT1 by RSV has been shown to depend on the fluorophore-labelled assay substrate and may disappear when native substrates are used. Claims that an RSV-alginate formulation acts primarily through direct SIRT1 activation should therefore be qualified and supported by orthogonal assays using native substrates, pathway perturbation, and downstream target engagement. Similarly, the antioxidant or pro-oxidant behavior of RSV depends on concentration, exposure duration, redox-active metals, cellular antioxidant capacity, and disease context [163]. Formulation studies should relate released and intracellular RSV concentrations to ROS generation, mitochondrial injury, apoptosis, and normal-cell toxicity rather than assuming a uniformly antioxidant mechanism.

Standardized reporting would substantially improve comparability. At minimum, future studies should distinguish technical from biological replicates, report the number of independently manufactured batches, provide complete release profiles rather than selected time points, quantify both local efficacy and systemic exposure, and include an appropriate free-RSV and unloaded-hydrogel control. For oncology, pharmacokinetic-pharmacodynamic analysis should connect local release to intratumoral RSV exposure and mechanistic thresholds; for wound healing, closure measurements should be supported by histological and functional outcomes. These measures would allow reproducibility and translational readiness to be evaluated alongside efficacy.

### Current clinical status, commercial landscape, and path forward

Commercial alginate wound dressings provide clinically established precedents for the development of RSV–alginate products. AlgiSite M is a calcium-alginate non-woven dressing cleared by the FDA under 510(k) K983210 for moderately to heavily exuding partial- and full-thickness wounds. Kaltostat, represented by FDA 510(k) K904488, is a mixed calcium–sodium alginate dressing that also supports the management of minor bleeding at donor sites and superficial wounds. Both products form a soft gel upon contact with wound exudate and establish practical benchmarks for fluid handling, conformability, wound contact, and clinical usability.

Clinical evidence for RSV-containing hydrogels remains limited. In a single-blind, split-face pilot study involving 20 patients with acne vulgaris, a carboxymethylcellulose-based RSV hydrogel applied for 60 days reduced clinical severity and microcomedone area without reported treatment-related adverse effects [164]. However, the small sample size and non-alginate matrix limit its relevance to RSV-loaded alginate systems. NCT06521333 subsequently compared a resveratrol-based gel with 0.2% hyaluronic acid gel and an acrylic palatal stent in 30 participants with palatal donor-site wounds [165], and the available formulation details were insufficient to identify it as an alginate-based hydrogel. Thus, RSV-loaded alginate hydrogels remain at the preclinical stage, without established clinical efficacy or regulatory approval.

The commercial and regulatory history of AlgiSite M and Kaltostat demonstrates the feasibility of bringing alginate-based wound-contact materials into routine clinical and commercial use. Incorporation of RSV and associated therapeutic claims could alter the product’s intended use and regulatory identity. The applicable pathway would depend on the primary mode of action, presentation, administration route, and proposed claims of the final formulation. Existing dressings provide material, manufacturing, and in-use performance baselines, whereas an RSV-containing formulation would require additional controls for drug identity, potency, release, stability, pharmacokinetics, and therapeutic efficacy.

Commercialization will require a product configuration that remains manufacturable, stable, usable, and economically viable beyond laboratory scale. Major cost drivers are likely to include highly characterized alginate, pharmaceutical-grade RSV, analytical control of RSV isomerization and degradation products, aseptic processing or validated sterilization, light- and oxygen-protective packaging, and long-term stability testing. A lyophilized or two-component kit may reduce storage and transportation constraints, although reconstitution time, mixing consistency, dose uniformity, and user handling would become additional development requirements. Early collaboration with a contract development and manufacturing organization experienced in combination products should address scale-dependent mixing and gelation, release specifications, container–closure compatibility, extractables and leachables, cost of goods, intellectual-property position, reimbursement, and technology transfer between manufacturing sites. The target product profile should define the intended population, administration setting, dosing frequency, storage conditions, clinical comparator, and minimum clinically meaningful efficacy advantage before expensive scale-up begins.

Adjacent local drug-delivery products provide useful benchmarks for regulatory and clinical development. Zusduri, a mitomycin intravesical formulation developed as UGN-102, demonstrates that a locally retained drug formulation can progress to regulatory approval when its manufacturing controls, target population, dosing procedure, and clinical benefit are clearly defined. OncoGel completed early Phase I/II clinical evaluation and its development ended before regulatory approval, illustrating the additional evidentiary requirements associated with late-stage clinical development [166]. Alginate-based injectable and implantable products, including Gut Guarding Gel evaluated under NCT03321396 and the ventricular-support hydrogel Algisyl, have also entered human evaluation. These products provide broader clinical-development and regulatory precedents while RSV-specific pharmacological and clinical questions remain unresolved [160].

Recent advances in adjacent hydrogel and nanofiber technologies provide contemporary design benchmarks for the next generation of RSV–alginate systems. MXene-based stimuli-responsive hydrogels illustrate the integration of electrical and electrochemical responsiveness into multifunctional wound materials [167]. Bioinspired polyurethane composite fibers capable of releasing nitric oxide and bioactive peptides demonstrate the delivery of multiple therapeutic signals from a single polymeric matrix and suggest a possible framework for combining RSV with vascular-regulatory or pro-angiogenic agents [168]. Digital-light-processing bioprinting enables more deterministic fabrication of hydrogel pore architecture and may support improved manufacturing reproducibility and cellular infiltration [169]. Protein-based hydrogels incorporating dual dynamic crosslinking provide a model for coordinating nerve repair, angiogenesis, and tissue remodeling in diabetic wounds and may inform the development of mechanically adaptable alginate–RSV networks [170]. Zwitterionic superlubricated nano-skins demonstrate how durable surface hydration and low-friction interfaces can improve postoperative anti-adhesion performance, with potential relevance to implantable or surgical-site RSV–alginate formulations [171]. Lubricative lecithin-based electrospun nanofibers provide a more recent extension of this surface-engineering strategy [172].

Further opportunities include the use of clinically established excipients, additive manufacturing of patient-specific dressings for irregular wound geometries, and incorporation of validated pH- or redox-responsive indicators for monitoring the wound microenvironment and formulation performance. Each added function should address a predefined critical quality attribute, manufacturing requirement, or clinically relevant endpoint. Two-dimensional-material reinforcement, nitric-oxide co-delivery, photo-patterned pore architectures, dual dynamic crosslinking, and lubricative surface engineering can be considered when their benefits justify the associated analytical, toxicological, manufacturing, and regulatory requirements. A credible translational strategy should prioritize product stability, batch reproducibility, scalable manufacturing, and measurable clinical benefit, with formulation complexity aligned to clearly defined therapeutic objectives.

## Conclusion and future perspectives

The pursuit of intelligent, multifunctional materials capable of advancing therapeutic and diagnostic practice has long motivated innovation in the biomedical materials field. Within this context, hydrogels have established themselves as compelling delivery vehicles for both small-molecule drugs and macromolecular biologics, while concurrently offering an ECM-like microenvironment that approximates the three-dimensional cellular niche. Alginate-based networks, in particular, have been studied in depth across a wide spectrum of biomedical uses. Achieving clinical viability for these systems, however, demands careful control over several interdependent variables, including polymer and crosslinker concentrations, network mesh dimensions, porosity, and overall crosslink density.

This article has provided a thorough overview of RSV-laden sodium alginate hydrogels in the contexts of oncology and wound repair. Pairing alginate as the structural scaffold with RSV as the bioactive payload represents a rational and economically attractive strategy for both therapeutic domains. Beyond its natural abundance, alginate offers durable stability, good cytocompatibility, and ready amenability to chemical functionalization. RSV, for its part, contributes well-documented antitumor, anti-inflammatory, antioxidant, and pro-regenerative activities relevant to tissue repair. Rational design of effective RSV-alginate hydrogels for specific indications requires a firm grasp of how these constructs behave once exposed to biological environments; accordingly, this manuscript has examined in detail the molecular targets, cellular responses, and intracellular trajectory of RSV, and has also analyzed both intrinsic network parameters and extrinsic stimuli that govern drug release from the hydrogel matrix.

Nonetheless, several obstacles persist. Chemical crosslinking agents commonly employed for alginate network formation must be thoroughly purged prior to biomedical deployment, and fine-tuning the crosslinking density remains technically challenging. Alginate matrices additionally suffer from relatively modest mechanical performance, although this limitation can be mitigated through reinforcement with nanoparticulate fillers, auxiliary polymers, or alternative crosslinkers. A further barrier to clinical translation is the absence of intrinsic cell-adhesion motifs on the alginate backbone. Functionalizing the matrix with exogenous molecules is similarly non-trivial, since reaction conditions must be carefully controlled to avoid the polymer degradation that occurs under strongly acidic or alkaline environments. Equally pressing is the need to secure consistent, high-grade alginate raw material with defined molecular architecture, molecular weight distribution, and reproducible crosslinking behavior—factors that will become increasingly critical as these technologies approach regulatory evaluation and clinical deployment.

The poor aqueous solubility and light sensitivity of RSV likewise demand attention. Encapsulation conditions exert a decisive influence on the compound’s release kinetics and storage stability within the alginate network. One promising route for overcoming the shortcomings of chemically crosslinked alginate lies in the substitution of conventional crosslinkers with cell-cleavable peptide linkers that enable enzyme-triggered release. Lowering crosslink density and correlating this with mechanical response represents another avenue worth systematic investigation. Co-encapsulation of RSV with complementary bioactives or inorganic nanomaterials offers an additional means of bolstering mechanical behavior while delivering synergistic therapeutic action. Ultimately, realizing the full clinical potential of RSV-loaded alginate hydrogels will benefit from an interdisciplinary effort that unites materials science, pharmacology, and translational medicine to enable diverse biomedical applications.

## Data Availability

No datasets were generated or analysed during the current study.
